# Assessing the association between smoking and hypertension: Smoking status, type of tobacco products, and interaction with alcohol consumption

**DOI:** 10.3389/fcvm.2023.1027988

**Published:** 2023-02-09

**Authors:** Ningxin Gao, Tao Liu, Yawen Wang, Min Chen, Lisha Yu, Chaowei Fu, Kelin Xu

**Affiliations:** ^1^Ministry of Education Key Laboratory of Public Health Safety, Department of Biostatistics, School of Public Health, Fudan University, Shanghai, China; ^2^Guizhou Province Center for Disease Control and Prevention, Guiyang, Guizhou, China; ^3^Ministry of Education Key Laboratory of Public Health Safety, Department of Epidemiology, School of Public Health, Fudan University, Shanghai, China

**Keywords:** tobacco use, hypertension, dose-response association, cohort study, interaction effect

## Abstract

**Background:**

The association between tobacco use and hypertension risk has been extensively researched but remains controversial, and few existing studies have considered the role of tobacco type and dosage response in this association. In this context, this study aims to provide epidemiological evidence for the possible relationship between tobacco smoking and future hypertension risk, with the tobacco type and consumption dose into consideration.

**Methods:**

This study was based on 10-year follow-up data from the Guizhou Population Health Cohort conducted in southwest China. Multivariate Cox proportional hazards regression models were used to estimate hazard ratios (HRs) and 95% confidence intervals [95% confidence intervals (CIs)], and restricted cubic spline analyses were performed to visualize the dose-response association.

**Results:**

A total of 5,625 participants (2,563 males and 3,062 females) were included in the final analysis. Heavy smokers of machine-rolled cigarettes had an elevated hypertension risk compared with non-smokers (HR: 1.50, 95% CI: 1.05–2.16). The interaction effects of heavy smoking-heavy drinking patterns increased the future hypertension risk, with an adjusted HR of 2.58 (95% CI: 1.06–6.33).

**Conclusion:**

This study did not find a significant association between overall tobacco use status and the risk of hypertension. However, heavy machine-rolled cigarette smokers had a statistically significant increased risk of hypertension compared with non-smokers, and a J-shape association has been found between the average daily consumption of machine-rolled cigarettes and the risk of hypertension. Besides, tobacco and alcohol consumption jointly increased the long-term hypertension risk.

## 1. Introduction

Hypertension is one of the major public health issues facing the world, causing substantial diseases and premature deaths globally (1). China accounts for a considerable share of the global burden of hypertension morbidity and mortality (2–4). Recent epidemiological research in mainland China indicated that nearly half of middle-aged Chinese suffered from hypertension, with a controlled rate of less than one in twelve (5). Several modifiable lifestyle factors have been considered a risk for hypertension and are receiving increasing attention. As a common and modifiable lifestyle risk factor, tobacco use is responsible for more than eight million deaths annually (6), resulting in significant public health and socio-economic burdens (7, 8).

However, the impact of tobacco use on the risk of future hypertension remains inconclusive. Unlike acute tobacco smoking exposure, which can induce transient sympathetic hypertensive effects (9), the influence of chronic exposure to tobacco smoking on hypertension appears to be complex and somewhat mixed. Some research found that tobacco use increases the risk of developing hypertension (10–12), but other epidemiological studies have shown that current smoking is linked to similar or lower blood pressure levels (13, 14). Notably, in these aforementioned studies, information about tobacco products was unclear or even missing, which may expose these studies to potentially confounding interference. In addition, existing studies recognize that smoking and alcohol consumption had synergistic and negative effects on cardiovascular function (15, 16), resulting in an increased risk of multiple diseases and adverse outcomes. Several studies have documented a significant increase in blood pressure or hypertension risk due to the tobacco-alcohol interaction (12, 17, 18), but to our knowledge, none of them has quantificationally assessed the interaction effect between alcohol and tobacco consumption on hypertension risk.

Therefore, based on a prospective cohort study in southwest China, we investigate the long-term effect of tobacco smoking on future hypertension risk, considering the types and consumption amount of tobacco. Besides, we further investigate the effects of tobacco-alcohol interaction on future hypertension and quantified the risk according to tobacco and alcohol consumption.

## 2. Materials and methods

### 2.1. Study design

The Guizhou Population Health Cohort was a community-based prospective cohort in Guizhou province, China. The baseline survey was conducted between 2010 and 2012, which comprised 9,280 participants from 48 townships of 12 districts (or counties) in Guizhou province by multi-stage proportional stratified cluster sampling. Residents over the age of 18 who live locally and have no plans to move out are included in our admission criteria. All participants were followed up for onset hypertension and vital status through a follow-up survey from 2016 to 2020, and 1,117 were lost to follow-up (loss to follow-up rate: 12.04%). After excluding 2,132 participants with unknown baseline blood pressure and 406 participants with incomplete information on hypertension in the follow-up survey, 5,625 participants were included in the analysis, with an average follow-up time of 6.98 years.

The Institutional Review Board of Guizhou Province Center for Disease Control and Prevention approved this study (No. S2017-02). All subjects provided a written informed consent form at the time of registration.

### 2.2. Information on tobacco use

Information on tobacco use was collected through face-to-face interviews by uniformly trained investigators. Participants were first asked to respond to their current tobacco use status in the baseline survey, and current smokers were further questioned about the type of tobacco products they used, including machine-rolled cigarettes, hand-rolled cigarettes, pipe tobacco, cigar, and others. If participants answered “yes” to one or several tobacco products, the smoking frequency (cigarettes/day or week; grams/day or week) of each product was further collected. To analyze the independent effects of each type of tobacco product on the risk of hypertension, we further obtained subsets of exclusive users of machine-rolled cigarettes, hand-rolled cigarettes, and pipe tobacco. We calculated the average daily consumption of each kind of tobacco product. Divide weekly consumption by seven to acquire the daily consumption of each participant. Besides, exclusive users of machine-rolled cigarettes were further classified into three levels based on the average daily consumption: mild smokers (less than 10 cigarettes/day), moderate smokers (10–20 cigarettes/day), and heavy smokers (more than 20 cigarettes/day) (17).

### 2.3. Ascertainment of hypertension

Blood pressure measurement was performed by trained investigators using uniformly calibrated electronic sphygmomanometers, accurate to 0.1 mmHg. Hypertension was defined if participants met any of the following criteria: (1) self-reported physician-diagnosed hypertension; (2) use of anti-hypertensive medications; (3) systolic blood pressure (SBP) ≥ 140 mmHg and/or diastolic blood pressure (DBP) ≥ 90 mmHg (19).

### 2.4. Covariates

Baseline covariates, including sex, age, area, ethnicity, occupation, marital status, physical activity, history of diabetes, and alcohol use status, were collected by interview. Area was divided into urban and rural, and ethnicity was grouped into Han nationality and other ethnic minorities. Occupation was classified into three categories: farming, unemployed or retired, and other. Marital status was classified as married, unmarried, and other. Physical activities were divided into sport and inactivity. Participants with type 2 diabetes (T2DM) at baseline or previously were considered to have a history of diabetes. Criteria for type 2 diabetes and physical activity have been reported in the previous literature (20). Height and weight of each participant were collected by trained physicians with an accuracy of 0.001 m and 0.1 kg, respectively. Body mass index (BMI) was calculated as body weight in kilograms divided by the square height in meters (kg/m^2^). The venous blood of participants was sampled after fasting for at least 8 h to obtain the blood biochemical indicators, including total cholesterol (TC), triglyceride (TG), high-density lipoprotein cholesterol (HDL-C) and low-density lipoprotein cholesterol (LDL-C). Based on the questionnaire, we calculated the average daily ethanol intake of the participants as described in the previous report (20), and divided participants into four groups: non-drinking (0 g/d), light drinking (0–12 g/d), moderate drinking (12–24 g/d), heavy drinking (> 24 g/d).

### 2.5. Statistical analysis

Student’s *t*-test and the chi-square test were used to compare the baseline characteristics of participants across groups. Multivariate Cox proportional hazards regression model was used to calculate the hazard ratios (HRs) and 95% confidence interval [95% confidence intervals (CIs)] of smoking indicators on the risk of incident hypertension. We tested the proportional hazards assumption and established time-related covariates for variables that failed the PH assumption. Three independent models were used in the analysis. In Model 1, no variables were adjusted. In Model 2 we adjusted for age and sex. In Model 3, we further adjusted for area, ethnicity, marital status, occupation, alcohol use, physical activity, history of diabetes, SBP, TC, triglycerides, HDL-C value, LDL-C value, and baseline BMI value. Restricted cubic spline analyses were applied to characterize the dose-response relationship and explore the potential non-linear association between the average daily consumption of each type of tobacco product with hypertension risk. In addition, the interaction effect between smoking and other covariates was also performed. Tests for interaction effect were conducted with multivariate-adjusted Cox proportional hazards regression analyses by adding the product of smoking and other covariates in models.

All data of this study were analyzed with R software (version 4.1.0). All statistical tests were two-sided, and the statistical significance was *P* < 0.05.

## 3. Results

### 3.1. Baseline characteristics

Table 1 showed the baseline characteristics of 5,625 participants. A total of 1,216 new hypertension cases were identified during follow-up, with an incidence of 21.62%. As shown in Table 1, compared with participants who did not develop hypertension at the follow-up survey, newly identified hypertension cases tended to be older, more likely to be male, more likely to be rural, more like to be Han Chinese, and had a higher baseline BMI, triglycerides, and SBP level. Statistically significant differences were also found in marital status, occupation, and current smoking status across groups. In addition, we described the baseline characteristics of participants grouped by smoking status at baseline, and the results are presented in Supplementary Table 1.

**TABLE 1:** Baseline characteristics according new-onset hypertension.

	Total	New-onset hypertension		*P*-value
		No	Yes	
Participants, *n*	5,625	4,409	1,216	
**Basic indicators**				
Rural, %	3,764 (66.9)	2,921 (66.3)	843 (69.3)	0.005
Age at baseline, years	42.03 ± 14.17	40.60 ± 13.85	47.23 ± 14.13	<0.001
Male, %	2,563 (45.6)	1,972 (44.7)	591 (48.6)	0.018
Ethnic minority, %	2,389 (42.5)	1,905 (43.2)	484 (39.8)	0.036
Marriage, %				<0.001
Married	4,532 (80.6)	3,526 (80.0)	1,006 (82.7)	
Unmarried	609 (10.8)	531 (12.0)	78 (6.4)	
Others	484 (8.6)	352 (8.0)	132 (10.9)	
Occupation, %				<0.001
Farmer	3,205 (57.0)	2,449 (55.5)	756 (62.2)	
Others	1,594 (28.3)	1,306 (29.6)	288 (23.7)	
Unemployed or retired	826 (14.7)	654 (14.8)	172 (14.1)	
Smoking, %	1,516 (27.0)	1,151 (26.1)	365 (30.0)	0.007
Alcohol use, %	1,737 (30.9)	1,355 (30.7)	382 (31.4)	0.674
Physical activity, %	4,861 (86.4)	3,805 (86.3)	1,056 (86.8)	0.660
BMI, kg/m^2^*	22.51 ± 3.16	22.42 ± 3.15	22.86 ± 3.17	<0.001
SBP, mmHg*	116.20 ± 11.94	115.36 ± 11.87	119.25 ± 11.70	<0.001
History of diabetes, %*	355 (6.3)	269 (6.1)	86 (7.1)	0.055
**Biochemical indicators**				
Triglycerides, mg/dl*	1.65 ± 1.49	1.61 ± 1.45	1.79 ± 1.65	<0.001
Total cholesterol, mg/dl*	4.73 ± 1.30	4.71 ± 1.31	4.77 ± 1.25	0.173
HDL cholesterol, mg/dl*	1.45 ± 0.57	1.45 ± 0.55	1.46 ± 0.63	0.913
LDL cholesterol, mg/dl*	2.62 ± 1.17	2.62 ± 1.17	2.59 ± 1.15	0.396

*Missing value.

BMI, body mass index; SBP, systolic blood pressure; HDL cholesterol, high-density lipoprotein cholesterol; LDL cholesterol, low-density lipoprotein cholesterol.

### 3.2. Associations between baseline smoking and incident hypertension

As shown in Table 2, the future hypertension risk did not differ significantly between current tobacco users and non-smokers (Model 3). For machine-rolled cigarette smokers, the heavy smoking pattern increased the risk of hypertension compared with non-smoking (HR: 1.50, 95% CI: 1.05–2.16). However, pure smokers of hand-rolled cigarettes and pipe tobacco shared a similar hypertension risk with non-smokers.

**TABLE 2:** Hazard ratios (95% confidence intervals) of hypertension associated with smoking status.

	Cases, *n*	HR (95% CI)		
		Model 1	Model 2	Model 3
**Smoking status at baseline**				
No	4,109	1.00	1.00	1.00
Yes	1,516	1.17 (1.03, 1.32)*	1.05 (0.89, 1.23)	0.97 (0.82, 1.15)
**Machine-rolled cigarettes**				
No	4,109	1.00	1.00	1.00
Light	520	1.04 (0.86, 1.26)	1.03 (0.83, 1.28)	0.99 (0.79, 1.25)
Moderate	697	1.03 (0.87, 1.23)	0.98 (0.80, 1.20)	0.93 (0.75, 1.14)
Heavy	108	1.72 (1.23, 2.42)**	1.51 (1.06, 2.16)*	1.50 (1.05, 2.16)*
**Hand-rolled cigarettes**				
No	4,109	1.00	1.00	1.00
Yes	34	1.12 (0.58, 2.16)	0.69 (0.36, 1.35)	0.66 (0.33, 1.29)
**Pipe tobacco (time < 6.4 years)**				
No	1,462	1.00	1.00	1.00
Yes	54	2.52 (1.75, 3.63)***	1.36 (0.91, 2.06)	1.15 (0.71, 1.86)
**Pipe tobacco (time ≥ 6.4 years)**				
No	2,647	1.00	1.00	1.00
Yes	48	1.50 (0.80, 2.80)	0.67 (0.35, 1.29)	0.72 (0.38, 1.40)

Model 1: no variables were adjusted.

Model 2: adjusted for age (continuous variable) and sex.

Model 3: model 2 plus area, ethnicity, marriage, occupation, alcohol use, physical activity, history of diabetes, SBP, total cholesterol, triglycerides, HDL-C value, LDL-C value, and baseline BMI value.

***P < 0.001, **P < 0.01, *P < 0.05.

HR, hazard ratio; 95% CI, 95% confidence interval.

We also examined the potential non-linear relationship between the average daily consumption of each type of tobacco with incident hypertension. The non-linear test indicated a significant non-linear relationship between the average daily consumption of machine-rolled cigarettes and the future hypertension risk (*P* for non-linear trend < 0.001), and a significant non-linear relationship was also found in exclusive users of pipe tobacco between the average consumption of tobacco products and future hypertension risk. No such relationships were found in hand-rolled cigarettes (*P* > 0.05).

**FIGURE 1:**
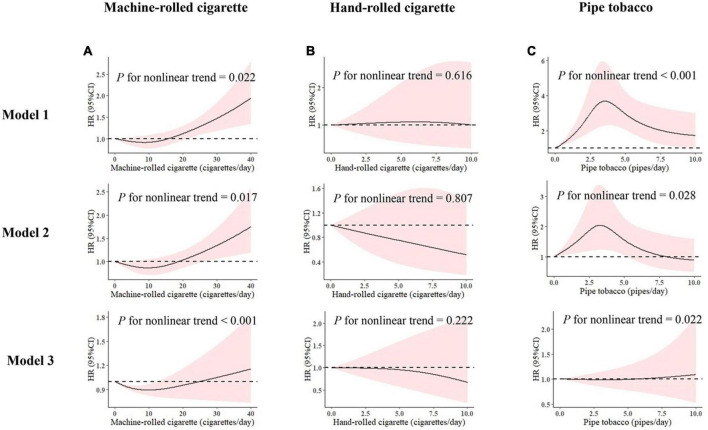
Multivariable adjusted hazard ratios with 95% CI for the association of average daily consumption of **(A)** machine-rolled cigarettes, **(B)** hand-rolled cigarettes, and **(C)** pipe tobacco with 0 as the reference value. Model 1: no variables were adjusted. Model 2: adjusted for age (continuous variable) and sex. Model 3: model 2 plus area, ethnicity, marriage, occupation, alcohol use, physical activity, history of diabetes, SBP, total cholesterol (TC), triglycerides, HDL-C value, LDL-C value, and baseline body mass index (BMI) value. HR, hazard ratio; 95% CI, 95% confidence interval.

Multivariate restricted cubic spline analyses suggested a J-shape association between the average daily consumption of machine-rolled cigarettes and the risk of hypertension (Figure 1A). A slight downward trend was observed at lower levels of smoking, and the risk curve then bottomed out at around 10 cigarettes/day and gradually rose among exclusive machine-rolled cigarette users. For pure hand-rolled cigarettes, the risk decreased slowly as average daily consumption increased (Figure 1B). For pipe tobacco, we found that the risk of hypertension increased rapidly and then decreased with growing average daily consumption without adjustment, and with the adjustment only for sex and age. While a smooth and slight upward curve was observed with increasing daily consumption grew after multivariate adjustment (Figure 1C).

### 3.3. The interaction effect between smoking and other covariates on hypertension

We further explored the interaction effect between tobacco consumption status and other covariates on hypertension and observed that the interaction between drinking and smoking status significantly affected the future hypertension risk. As shown in Table 3, participants who smoke and drink heavily at the same time had a higher chance of developing hypertension (HR: 1.45, 95% CI: 1.08–1.95). Considering the interaction effects of smoking and drinking, we further explore the interaction effect on hypertension by establishing a new model containing the tobacco-alcohol interaction term in Supplementary Table 2.

**TABLE 3:** Effect of interaction with smoking on the incidence of hypertension.

	HR (95% CI)		
	Model 1	Model 2	Model 3
Sex: smoking	1.08 (0.57, 2.07)	1.23 (0.64, 2.34)	1.47 (0.74, 2.90)
Area: smoking	0.80 (0.61, 1.03)	0.85 (0.65, 1.10)	0.81 (0.62, 1.05)
Ethnicity: smoking	1.05 (0.81, 1.36)	1.01 (0.78, 1.31)	1.01 (0.78, 1.32)
Occupation: smoking	0.82 (0.56, 1.19)	0.74 (0.51, 1.07)	0.71 (0.49, 1.04)
Physical activity: smoking	1.20 (0.80, 1.79)	0.97 (0.65, 1.45)	1.02 (0.68, 1.53)
Alcohol use: smoking	1.21 (0.91, 1.60)	1.36 (1.02, 1.82)*	1.45 (1.08, 1.95)*
Age: smoking	1.00 (1.00, 1.01)	1.00 (1.00, 1.01)	1.00 (0.99, 1.01)
Marriage: smoking	0.75 (0.60, 0.93)**	0.89 (0.73, 1.10)	0.91 (0.74, 1.12)
BMI: smoking	1.00 (0.96, 1.04)	1.00 (0.96, 1.04)	1.00 (0.96, 1.05)
SBP: smoking	0.99 (0.98, 1.00)	0.99 (0.98, 1.01)	1.00 (0.99, 1.01)
Triglycerides: smoking	1.04 (0.96, 1.15)	1.06 (0.97, 1.17)	1.05 (0.96, 1.16)

Model 1: no variables were adjusted.

Model 2: adjusted for age (continuous variable) and sex.

Model 3: model 2 plus area, ethnicity, marriage, occupation, alcohol use, physical activity, history of diabetes, SBP, total cholesterol, triglycerides, HDL-C value, LDL-C value, and baseline BMI value.

**P < 0.01, *P < 0.05.

HR, hazard ratio; 95% CI, 95% confidence interval.

To further investigate the potential impact of this interaction on hypertension risk, we quantified the degree of risk according to the classification of alcohol and tobacco (machine-rolled cigarettes) consumption. As shown in Table 4, participants who drink heavily and smoke heavily exhibited a significantly elevated risk compared with non-smokers and non-drinkers, with an adjusted HR of 2.58 (95% CI: 1.06–6.33).

**TABLE 4:** Hazard ratios (95% confidence intervals) of hypertension associated with smoking and alcohol consumption.

	Average daily consumption of cigarettes (machine-rolled cigarette)				
		No	Light	Medium	Heavy
Average daily consumption of ethanol	**Model 1**				
	No	1.00	0.93 (0.67, 1.29)	0.96 (0.73, 1.28)	1.39 (0.76, 2.52)
	Light	0.88 (0.70, 1.10)	1.10 (0.82, 1.48)	0.84 (0.61, 1.16)	1.19 (0.53, 2.67)
	Medium	1.33 (0.88, 1.99)	1.17 (0.69, 1.99)	1.16 (0.73, 1.86)	1.81 (0.75, 4.36)
	Heavy	1.72 (1.13, 2.63)*	1.08 (0.66, 1.75)	1.41 (1.03, 1.93)*	2.87 (1.66, 4.97)***
	**Model 2**				
	No	1.00	0.91 (0.65, 1.29)	0.87 (0.65, 1.18)	1.15 (0.63, 2.11)
	Light	0.89 (0.71, 1.12)	1.19 (0.87, 1.65)	0.85 (0.60, 1.19)	0.99 (0.44, 2.22)
	Medium	1.17 (0.77, 1.77)	1.08 (0.63, 1.85)	1.13 (0.70, 1.83)	1.71 (0.71, 4.16)
	Heavy	1.39 (0.90, 2.14)	0.96 (0.58, 1.56)	1.32 (0.94, 1.84)	2.94 (1.67, 5.15)***
	**Model 3**				
	No	1.00	0.86 (0.60, 1.22)	0.82 (0.60, 1.12)	1.25 (0.68, 2.30)
	Light	0.79 (0.38, 1.66)	1.14 (0.53, 2.44)	0.76 (0.35, 1.66)	0.87 (0.30, 2.53)
	Medium	1.06 (0.47, 2.38)	0.96 (0.40, 2.34)	0.95 (0.41, 2.21)	1.51 (0.49, 4.68)
	Heavy	1.25 (0.55, 2.82)	0.82 (0.35, 1.95)	1.20 (0.55, 2.58)	2.58 (1.06, 6.33)*

Model 1: no variables were adjusted.

Model 2: adjusted for age (continuous variable) and sex.

Model 3: model 2 plus area, ethnicity, marriage, occupation, alcohol use, physical activity, history of diabetes, SBP, total cholesterol, triglycerides, HDL-C value, LDL-C value, and baseline BMI value.

***P < 0.001, *P < 0.05.

HR, hazard ratio; 95% CI, 95% confidence interval.

## 4. Discussion

Based on 5,625 eligible adult participants in the Guizhou Population Health Cohort, we analyzed the risk of hypertension among different tobacco product users and explored the dose-response effects of these tobacco products. The novel and principal finding of this research was that heavy machine-rolled cigarette users are at a greater risk for future hypertension compared with those people who do not use tobacco products. Additionally, our results support the idea that tobacco and alcohol can jointly contribute to an increased risk of hypertension, especially in heavy drinking and heavy smoking population. The findings provided longitudinal evidence for the effect of using different types of tobacco products on hypertension risk and indicated the significance of tobacco-alcohol interaction tobacco on hypertension prevention, suggesting important public health practice implications.

Although tobacco use is widely perceived as an important risk factor for cardiovascular impairment and multiple chronic non-communicable diseases, the relationship between smoking and hypertension is still complex to be elucidated. Some previous studies have reported that current tobacco users have a similar or even lower hypertension risk compared with non-smokers (21–23). In contrast, other research has observed an elevated risk of future hypertension among current tobacco users compared with non-smokers (24–27). The discrepancy in these results may be partly attributed to the complicated effect of tobacco use on blood pressure regulation and inadequate adjustment for confounders. After adjusting for covariates affecting blood pressure, our findings were in line with several previous research, suggesting that overall tobacco use leads to a similar risk of future hypertension (16, 28).

Previous research has found that different types of tobacco products produce different health effects (29–31). However, insufficient attention has been paid to the potential differences in the effect of tobacco product types on blood pressure, leading to a lack of research evidence. To fill this knowledge gap, we further analyzed the risk of hypertension among different types of tobacco products. Among machine-rolled cigarette users, a J-shape association between tobacco consumption and risk of hypertension was found, implying that light or moderate smokers may have a slightly, although not significantly, decreased risk of hypertension. However, heavy machine-rolled cigarette smokers carried a higher risk compared with non-smokers. A similar trend between tobacco consumption and blood pressure was observed among women in another survey, but this research lacked information on the type of tobacco products and only counted the number of cigarettes as an indicator of smoking (12). Nevertheless, a prospective cohort study of Japanese male workers indicated a positive correlative dose-effect association between the average consumption of cigarettes and the risk of developing hypertension (18). Considering that machine-rolled cigarettes are the most popular and common tobacco products, our findings further support that heavy smoking patterns can be detrimental to blood pressure control. An unanticipated finding was that the exclusive users of pipe tobacco and hand-rolled cigarette shared a similar risk of incident hypertension as non-smokers, as the dosage-response analysis showed that they have negative and insensitive relationships with the risk of onset hypertension. Despite the lack of studies on the relationship between hand-rolled cigarettes/pipe tobacco use on blood pressure, previous studies have indicated their risks for cardiovascular health (32, 33). Given the lack of similar studies and the small number of users of these two types of tobacco in our study, the relationship between these two types of tobacco consumption and hypertension needs to be further investigated.

Alcohol and tobacco use are highly comorbid, and they have been found to interact with each other, leading to more frequent use and higher consumption levels (34), which highlights the importance of paying attention to their co-effects of them on public health. In this study, we found that heavy smoking and heavy drinking patterns can act synergistically to increase hypertension risk. This finding was in line with previous research (17, 18). A possible mechanism for this phenomenon is that both chronic tobacco and alcohol consumption can generate a direct or indirect decrease in vascular compliance through endothelial damage and chronic inflammation (35, 36). Our findings suggest that the co-occurrence of heavy smoking and heavy drinking may pose multiplicative hypertension risks, emphasizing the importance of controlling the concurrence of heavy smoking and heavy alcohol consumption in blood pressure control.

Based on the prospective natural population cohort in Guizhou province, our research provides epidemiological evidence of hypertension risk among consumers of different types of tobacco products in a multiethnic population of southwest China. However, this study also has some limitations. First, the data on tobacco consumption were only obtained from the baseline survey, resulting in a lack of information on exposure duration. Meanwhile, smoking information was obtained through the interview survey, and the actual smoking status of the participants was not verified by the laboratory tests, which may lead to inaccurate information. Second, due to the insufficient times for follow-up visits and the large survey intervals, the recorded time of the onset of hypertension could be inaccurate. Third, although common confounding factors such as demographic characteristics, living habits, and biological indicators have been adjusted in our study, some potentially confounding factors may have been overlooked. Fourth, considering that hand-rolled and pipe tobacco are often produced on a small scale and lack industry standards, the quality of the two tobacco products is often inconsistent. Moreover, tobacco smokers rarely weigh and record the amount of tobacco carefully before smoking, which may result in certain information bias. Finally, considering the limitation of the sample size, the number of exclusive smokers of some tobacco products is inadequate, and further study is needed to further investigate the relationship between the consumption of those tobacco products and future hypertension.

## 5. Conclusion

This study found that exclusive heavy smokers of machine-rolled cigarettes had a significantly increased risk of hypertension compared with non-smokers. In addition, smoking and drinking can work together to significantly increase future hypertension risk. Our findings highlight that the type of tobacco products and the synergistic effect of tobacco and alcohol consumption on future hypertension should also be carefully considered. For the prevention of hypertension, limiting tobacco and alcohol consumption at the same time may yield better health outcomes in reducing the risk of hypertension.

## Data availability statement

The raw data supporting the conclusions of this article will be made available by the authors, without undue reservation.

## Ethics statement

The studies involving human participants were reviewed and approved by the Institutional Review Board of Guizhou Province Center for Disease Control and Prevention (No. S2017-02). The patients/participants provided their written informed consent to participate in this study.

## Author contributions

NG: formal analysis, methodology, and writing the manuscript. TL: funding acquisition, investigation, data curation, and methodology. YW: formal analysis, methodology, review, and editing the manuscript. MC and LY: funding acquisition, investigation, and data curation. CF and KX: formal analysis, methodology, investigation, supervision, review, and editing the manuscript. All authors contributed to the article and approved the submitted version.
