# Supplementary material for: Assessing the association between smoking and hypertension: Smoking status, type of tobacco products, and interaction with alcohol consumption
Source: Front Cardiovasc Med. 2023 Feb 9;10:1027988. doi: 10.3389/fcvm.2023.1027988 (PMC9947503; doi:10.3389/fcvm.2023.1027988)
Supplement: Supplementary file 1 [file Data_Sheet_1.PDF]

Supplementary Material

Assessing the Association between Smoking and Hypertension: Smoking Status, Type of Tobacco Products, and Interaction with Alcohol Consumption

Ningxin Gao[1†], Tao Liu[2†*], Yawen Wang[1], Min Chen[2], Lisha Yu[2], Chaowei Fu[3], Kelin Xu[1*]

[1] Department of Biostatistics, Ministry of Education Key Laboratory of Public Health Safety, School of Public Health, Fudan University, Shanghai, China

[2] Guizhou Province Centre for Disease Control and Prevention, 101 Bageyan Road, Yunyan District, Guiyang City, Guizhou Province, China

[3] Department of Epidemiology, Ministry of Education Key Laboratory of Public Health Safety, School of Public Health, Fudan University, Shanghai, China

†These authors contributed equally to this work and share first authorship

*** Correspondence:**
Kelin Xu
xukelin@fudan.edu.cn
or
Tao Liu
liutaombs@163.com

Supplementary Tables

sTable 1 Baseline characteristics according baseline smoking status

	Total	Baseline smoking status		*P value*
		No	Yes	
**Participants, n**	5625	4109	1516	
**New-onset hypertension**	1216 (21.6)	851 (20.7)	365 (24.1)	0.007
**Basic indicators**				
Rural, %	3764 (66.9)	2801 (68.2)	963 (63.5)	0.001
Age at baseline, years	42.03±14.17	41.84±14.27	42.56±13.89	0.089
Men, %	2563 (45.6)	1092 (26.6)	1471 (97.0)	<0.001
Ethnic minority, %	2389 (42.5)	1824 (44.4)	565 (37.3)	<0.001
Marriage, %				0.084
Married	4532 (80.6)	3322 (80.8)	1210 (79.8)	
Unmarried	609 (10.8)	423 (10.3)	186 (12.3)	
Others	484 (8.6)	364 (8.9)	120 (7.9)	
Occupation, %				<0.001
Farmer	3205 (57.0)	2304 (56.1)	901 (59.4)	
Others	1594 (28.3)	1120 (27.3)	474 (31.3)	
Unemployed or retired	826 (14.7)	685 (16.7)	141 (9.3)	
Alcohol use, %	1737 (30.9)	743 (18.1)	994 (65.6)	<0.001
Physical activity, %	4861 (86.4)	3510 (85.4)	1351 (89.1)	<0.001
BMI, kg/m^{2*}	22.51±3.16	22.58±3.22	22.33±2.98	0.007
SBP, mmHg*	116.20±11.94	115.40±12.09	118.38±11.25	<0.001
History of diabetes, %*	355 (6.3)	243 (5.9)	112 (7.4)	0.044
**Biochemical indicators**				
Triglycerides, mg/dL*	1.65±1.49	1.59±1.27	1.81±1.96	<0.001
Total cholesterol, mg/dL*	4.73±1.30	4.76±1.32	4.63±1.23	0.001
HDL cholesterol, mg/dL*	1.45±0.57	1.47±0.58	1.42±0.53	0.018
LDL cholesterol, mg/dL*	2.62±1.17	2.64±1.19	2.54±1.09	0.005

Note: * missing value.

Abbreviations: BMI, body mass index; SBP, systolic blood pressure; HDL cholesterol, high-density lipoprotein cholesterol; LDL cholesterol, low-density lipoprotein cholesterol.

sTable 2 Hazard ratios (95% confidence intervals) of hypertension associated with smoking status

	Cases, n	HR (95%CI)			
		**Model 1**	**Model 2**	**Model 3**	**Model4**
Smoking status at baseline					
No	4109	1.00	1.00	1.00	1.00
Yes	1516	1.17 (1.03, 1.32) *	1.05 (0.89, 1.23)	0.97 (0.82, 1.15)	0.81 (0.64, 1.02)
Machine-rolled cigarettes					
No	4109	1.00	1.00	1.00	1.00
Light	520	1.04 (0.86, 1.26)	1.03 (0.83, 1.28)	0.99 (0.79, 1.25)	0.85 (0.64, 1.13)
Moderate	697	1.03 (0.87, 1.23)	0.98 (0.80, 1.20)	0.93 (0.75, 1.14)	0.80 (0.61, 1.04)
Heavy	108	1.72 (1.23, 2.42) **	1.51 (1.06, 2.16) *	1.50 (1.05, 2.16) *	1.29 (0.87, 1.92)
Hand-rolled cigarettes					
No	4109	1.00	1.00	1.00	1.00
Yes	34	1.12 (0.58, 2.16)	0.69 (0.36, 1.35)	0.66 (0.33, 1.29)	0.55 (0.19, 1.60)
Pipe tobacco (Time<6.4 years)					
No	1462	1.00	1.00	1.00	1.00
Yes	54	2.52 (1.75, 3.63) ***	1.36 (0.91, 2.06)	1.15 (0.71, 1.86)	0.83 (0.51, 1.34)
Pipe tobacco (Time≥6.4 years)					
No	2647	1.00	1.00	1.00	1.00
Yes	48	1.50 (0.80, 2.80)	0.67 (0.35, 1.29)	0.72 (0.38, 1.40)	1.33 (0.89, 1.99)

Note: Model 1: no variables were adjusted.
Model 2: adjusted for age (continuous variable), sex.
Model 3: model 2 plus area, ethnicity, marriage, occupation, alcohol use, physical activity, and history of diabetes, SBP, total cholesterol, triglycerides, HDL-C value, LDL-C value, baseline BMI value.
Model 4: model 3 plus interaction term for tobacco and alcohol use.
***: $P<0.001$, **: $P<0.01$, *: $P<0.05$.
Abbreviations: HR, hazard ratio; 95%CI, 95% confidence interval.
